# Characterization of molecularly imprinted polymers for the extraction of tobacco alkaloids and their metabolites in human urine

**DOI:** 10.1002/bmc.5361

**Published:** 2022-04-26

**Authors:** Haley A. Mulder, Adam C. Pearcy, Matthew S. Halquist

**Affiliations:** ^1^ School of Pharmacy, Department of Pharmaceutics Virginia Commonwealth University Richmond Virginia USA

**Keywords:** cotinine, molecularly imprinted polymers, morphine, nicotine, solid‐phase extraction, tobacco‐specific nitrosamines

## Abstract

Molecularly imprinted polymers (MIPs) are synthetic polymers designed to selectively extract target analytes from complex matrices (including biological matrices). The literature shows that MIPs have a degree of cross‐selectivity from analytes within the same class of compounds. A commercially available MIP for tobacco‐specific nitrosamines (TSNAs) is designed to be class selective for four TSNA compounds. This study sought to characterize the extent of cross‐selectivity of the TSNA MIPs with other tobacco alkaloids. Cross‐selectivity and recovery of the SupelMIP™ TSNA SPE cartridges was assessed with *N*‐nitrosonornicotine (NNN), nicotine, cotinine and morphine. Their recoveries were compared with the recoveries of a nonimprinted polymer SPE cartridge, and two traditional SPE cartridges: a Waters mixed‐mode cation exchange cartridge and a Waters hydrophilic–lipophilic balance cartridge. NNN and cotinine had the highest recoveries with the MIP cartridge, over 80%, and cotinine samples in urine had >80% recoveries. Nicotine had highly variable recoveries, possibly owing to differing chemical properties from the TSNAs. All three analytes had significantly different recoveries with the MIP cartridges compared with the traditional SPE cartridges. Morphine displayed nonspecific interactions with the MIP cartridges. Utilization of the TSNAs’ cross‐selectivity allows for simultaneous extraction and identification of multiple tobacco biomarkers using one extraction technique.

## 1 INTRODUCTION

Molecularly imprinted polymers (MIPs) are synthetic polymers used to selectively extract target compounds from complex matrices (including biological matrices). They are created when a template analyte is copolymerized with a functional monomer and a cross‐linking monomer (Pichon et al., 2019). The cross‐linking monomer holds the functional monomer in place on the polymer, and the template analyte and functional monomer interact through noncovalent interactions, such as hydrogen bonding and electrostatic interactions (Turiel & Esteban, 2020). Post‐polymerization, the template is removed, leaving an imprint within the polymer, where the template analyte can selectively rebind with the functional monomer. The selective binding within the cavity allows for matrix interferences to be extensively washed and removed without disrupting the target analyte (Boyd et al., 2007; Hu et al., 2021). The literature reports that sample preparation with MIPs leads to favorable recoveries with low matrix effects, and without the need for extensive sample preparation often found with solid‐phase extraction (SPE) and liquid–liquid extraction (Hu et al., 2021).

The specificity and selectivity of the MIP are governed by the analyte's size, shape and functional chemistry, and its interactions with the functional monomer. Therefore, analytes with similar structures and chemical properties to the target analyte can be cross‐selective with the MIP (Hu et al., 2021; Yilmaz et al., 2015). The potential cross‐selectivity can be utilized for a class of compounds, instead of one, single compound. Murakami et al. demonstrated the advantages of the MIP cross‐selectivity. Using a MIP SPE cartridge designed for amphetamine, methamphetamine and 3,4‐methylnedioxymethamphetamine, 11 synthetic cathinones were successfully extracted from urine and whole blood. Through adjusting extraction parameters, such as the loading pH, the authors achieved recoveries of between 60 and 89% for the synthetic cathinones in urine. The favorable recoveries with the amphetamine‐MIP were compared with recoveries of 12–90% with hydrophilic‐based SPE and recoveries of 8–92% with liquid–liquid extraction. Further, the authors reported lower matrix effects with the amphetamine‐MIP cartridges compared with the traditional SPE method. The authors did note that as the analyte structure changed, specifically with the increase in an alkyl side chain, the recovery of the analyte diminished, indicating that the shape and size of the analyte does have an effect on the analyte's ability to interact with the imprinted cavity (Murakami et al., 2018).

Tobacco‐specific nitrosamines (TSNAs) are tobacco by‐products formed during the curing, harvesting and fermentation process of tobacco leaves (Stepanov & Hecht, 2005). Two TSNAs, *N*‐Nitrosonornicotine (NNN) and 4‐(methylnitrosamino)‐1‐(3‐pyridyl)‐1‐butanone (NNK), are highly carcinogenic (Hecht, 1998). TSNA levels are used as biomarkers of exposure to tobacco products in both smokers and nonsmokers, with concentrations <10 pg/ml in urine for nonsmokers. Owing to their low concentrations in urine, sensitive and reliable extraction techniques are required for accurate detection on analytical instruments (Boyd et al., 2007). Supelco has designed the commercial SupelMIP™ TSNA SPE cartridge that is “class selective” for four TSNAs: NNN, NNK, *N*‐nitrosonoranabasine, and *N*‐nitrosonoranatabine in urine, down to 4 pg/ml (SupelMIP SPE‐TSNA Data Sheet, n.d.). Previous studies have demonstrated that NNK's metabolite, 4‐(methylnitrosamino)‐1‐(3‐pyridyl)‐1‐butanol (NNAL) can also be extracted using the SupelMIP™ TSNA cartridge (Kavvadias et al., 2009; Xia et al., 2014). A SupelMIP™ NNAL SPE cartridge was also shown to be cross‐selective for the four TSNAs (Xia et al., 2005, 2014).

The current literature typically splits the tobacco exposure analysis to focus on nicotine, cotinine and *trans*‐3‐hydroxycotinine, or TSNAs and NNAL (Marques et al., 2021). Few papers have developed methods for the simultaneous detection of NNAL and cotinine in urine, but highlight the need for effective sample preparation methods to detect nonsmoker levels of the analytes without matrix interference (Kotandeniya et al., 2015; Luo et al., 2021). Since the TSNA MIP cartridge is designed to be class selective for the four TSNA analytes, and has demonstrated cross‐selectivity with NNAL, it is possible that the cross‐selectivity of the MIP can be expanded to include other tobacco alkaloids and their metabolites. This would allow for the measurement of total nicotine equivalence in one, simple extraction step.

This study sought to characterize the extraction capabilities of the TSNA MIP with other tobacco alkaloids: the TSNA precursor, nicotine, and nicotine's urinary metabolite, cotinine. The recoveries of NNN, nicotine and cotinine with a nonimprinted polymer (NIP) cartridge and two traditional SPE cartridges were compared with the recoveries with the TSNA MIP cartridge. Finally, morphine, an analyte that is of a different class of compounds than the tobacco alkaloids, was extracted with the four extraction techniques to determine the full extent of cross‐selectivity with the imprinted polymer.

## 2 MATERIALS AND METHODS

### 2.1 Materials and chemical reagents

SupelMIP™ TSNA (50 mg/3 ml) SPE cartridges were purchased from Sigma Aldrich. Oasis hydrophilic–lipophilic balance (HLB, 30 mg/1 ml) and mixed‐mode cation exchange (MCX, 30 mg/1 ml) SPE cartridges were purchased from Waters (Milford, MA, USA). The NIP material was bought from Biotage (Uppsala, Sweden).

HPLC grade acetonitrile, dichloromethane (DCM), heptane, methanol (MeOH), tetrahydrofuran (THF) and water (H_2_O) were purchased from VWR International (Radnor, PA). Ammonium formate (97%) and orthophosphoric acid (85% wt.) were purchased from Sigma Aldrich (St Louis, MO, USA) and ammonium acetate and triethyl amine (TEA) were purchased from Fisher Scientific (Waltham, MA, USA). Methyl *tert*‐butyrl ether (MtBE) was purchased from Honeywell (Charlotte, NC, USA). Cotinine (99.7%), cotinine‐d3 (99.8%), morphine (99.7%), nicotine (99.6%) and NNN (99,8%) were purchased from Cerilliant (Round Rock, TX, USA).

### 2.2 Preparation of reagents

Standard stock solutions of cotinine, morphine, nicotine and NNN were prepared individually at 100 μg/ml concentrations in MeOH and stored at −20°C. Standard working solutions were prepared fresh daily by diluting the analytes down to their appropriate concentrations in 10 mm ammonium acetate, at pH 5.5 or 9.2. The 10 mm ammonium acetate solutions were adjusted to their appropriate pH values using acetic acid or ammonium hydroxide and verified with a pH meter. Human urine was obtained from laboratory donations and stored at −20°C. Acidic levels of urine were verified using litmus paper. Samples were prepared fresh daily by diluting cotinine down to its appropriate concentrations in human urine.

### 2.3 SupelMIP™ TSNA MIP and NIP extraction protocol

Samples were extracted following a modified method recommended by the manufacturer (Table 1). The MIP cartridges were primed with 1 ml of methanol followed by 1 ml of water. The solvents were pulled through the cartridges by centrifugation. The protocol, including the revolutions per minute and time, are listed in Table 1. Samples were loaded onto the cartridge in 1 ml aliquots at 1 μg/ml concentrations in 10 mm ammonium acetate buffered to a pH of 5.5 or 9.2. The loading fraction was passed through the cartridge gravimetrically, and the cartridge was dried for 10 min to remove any residual aqueous solvent. A 1 ml aliquot of heptane was used to further remove any aqueous solvent and disrupt hydrophobic interactions. The cartridge was dried again, and the analytes were eluted with two 1 ml aliquots of 9:1 (v/v) DCM:MeOH. The wash and elution steps were evaporated at 55°C and reconstituted in 200 μl mobile phase. Nonimprinted polymer cartridges were created with nonimprinted polymer material purchased from Biotage. A frit was placed at the bottom of an emptied 3 ml SPE cartridge and 50 mg of the NIP material was slurry packed in a 1 ml aliquot of methanol. A second frit was placed above the NIP material and the same extraction protocol used for the MIP cartridges above was followed.

**TABLE 1:** Extraction protocol for tobacco‐specific nitrosamine molecularly imprinted polymer solid‐phase extraction cartridges

Step	Solvent	Revolutions per minute	Minutes
1. Prime	1 ml MeOH	200	3
	1 ml H_2_O	200	3
2. Load	1 ml sample in 10 mm ammonium acetate	Gravimetric	
3. Dry		1,500	10
4.Wash	1 ml heptane	700	6
5. Dry		1,500	2
6. Elute	1 ml 9:1 (v/v) DCM:MeOH	350	10
	1 ml 9:1 (v/v) DCM:MeOH	200	5

MeOH, Methanol; DCM, dichloromethane.

Cotinine in human urine was extracted using the SupelMIP™ TSNA cartridge. Cotinine at 10, 100 and 1,000 ng/ml concentrations was prepared in urine prior to the extraction. Cotinine‐d3 at 68 ng/ml was added to each sample and the sample underwent the SupelMIP™ TSNA SPE extraction outlined above.

### 2.4 Waters Oasis HLB and MCX extraction protocols

Samples were extracted following previously published methods for the extraction of NNN with a traditional SPE cartridge. In brief, the cartridges were primed with 1 ml aliquots of methanol followed by water. The same centrifuge parameters used for the MIP and NIP cartridges were used on the SPE cartridges. Samples were loaded onto the cartridge in 1 ml aliquots at 1 μg/ml. The samples extracted with the HLB cartridges were loaded under acidic conditions. NNN and cotinine were prepared in 10 mm ammonium formate, pH 2.73, and nicotine and morphine were prepared in 10 mm ammonium acetate, pH 5.5. The cartridges were washed with 1 ml of 95:5 (v/v) H_2_O:MeOH and eluted with two 1 ml fractions of methanol (Wu et al., 2003). The sample was evaporated at 55°C and concentrated in mobile phase. The samples extracted with the MCX cartridges were loaded under basic conditions at 10 mm ammonium acetate, pH 10, washed with 1 ml methanol and eluted with two 1 ml fractions of 9:1 (v/v) MeOH:25% NH_4_OH. Elution fractions were evaporated at 55°C and concentrated in mobile phase (Kavvadias et al., 2009).

### 2.5 HPLC apparatus

All experiments were conducted on a Waters Acquity H Class UPLC and a Waters Acquity PDA detector (Milford, MA, USA). For NNN and cotinine, chromatographic separation was carried out on a Phenomenex C_18_ column (50 × 2.1 mm, 2.6 μm) at 25°C. The aqueous mobile phase A was 10 mm ammonium acetate, pH 10, and the organic mobile phase B was acetonitrile under 90:10 aqueous–organic isocratic conditions. The flow rate was 0.3 ml/min and the PDA was set to monitor NNN at 238 nm and cotinine at 260 nm.

For nicotine, chromatographic separation was carried out on an Xterra RP_18_ column (150 × 4.6 mm, 5 μm) at 25°C. Mobile phase A was 0.1% (v/v) TEA in water pH adjusted to 7.6 ± 0.05 with orthophosphoric acid (85%) and 1 N NaOH. Mobile phases B and C were 0.1% (v/v) TEA in methanol and acetonitrile, respectively. Mobile phase D and diluent were 80% (v/v) methanol in water. The chromatographic conditions were operated under the following method: the quaternary pump was used with a gradient method with the initial mobile phase composition at 60:26:14 for mobile phases A–C, respectively, at a flow rate of 0.8 ml/min. At 6 minu, the gradient was changed to 100% mobile phase D and held for 2 min before returning to the initial conditions, which were held until the end of the run (15 min). The PDA was set to monitor nicotine at 260 nm (Gholap et al., 2018).

For morphine, chromatographic separation was carried out on the Xterra RP_18_ column (150 × 4.6 mm, 5 μm) at room temperature. Mobile phase A was 2 mm ammonium formate and mobile phase B was acetonitrile at 50:50 isocratic conditions. The flow rate was 0.8 ml/min and the PDA was set to monitor morphine at 210 nm (Chan, 2017).

### 2.6 LC–MS/MS apparatus

Recovery experiments were carried out on a Waters Alliance e2695 HPLC and a Waters Quattro Micro API mass spectrometer (Milford, MA, USA). Chromatographic separation was carried out with a Phenomenex synergi‐4 μ‐Polar RP column (150 × 4.6 mm, 4 μm) kept at 40°C. Aqueous mobile phase A was 10 mm ammonium acetate with 0.1% acetic acid in water and mobile phase B was 10 mm ammonium acetate with 0.1% acetic acid in methanol. The HPLC was kept at isocratic conditions of 60% mobile phase A at a flow rate of 0.65 ml/min.

The mass spectrometer was operated in positive ion mode using multiple reaction monitoring. The source block temperature was set to 150°C, the desolvation temperature was set to 450°C and the desolvation gas flow at 450 L/h. The capillary voltage, cone voltage, extractor and radio frequency (RF) lens were set to 2.85 kV, 30 V, 2 V and 0.4 V respectively. The entrance and exit lens were both set to 30 and the ion energy 0.6. The multiplier was set to 650 and the nebulizer gas flow was 150 L/h. Cotinine and cotinine‐d3 were quantified using the multiple reaction monitoring transition of [M + H]^+^ ion of cotinine at *m*/*z* 177.4 → 79.82 and cotinine‐d3 at *m*/*z* 180.4 → 79.93. The limit of detection and limit of quantitation were established based on signal‐to‐noise ratio of the lowest concentration injected. The limit of detection was determined to be 3 ng/ml and the limit of quantitation was 10 ng/ml.

## 3 RESULTS

### 3.1 *N*‐Nitrosonornicotine

**FIGURE 1:**
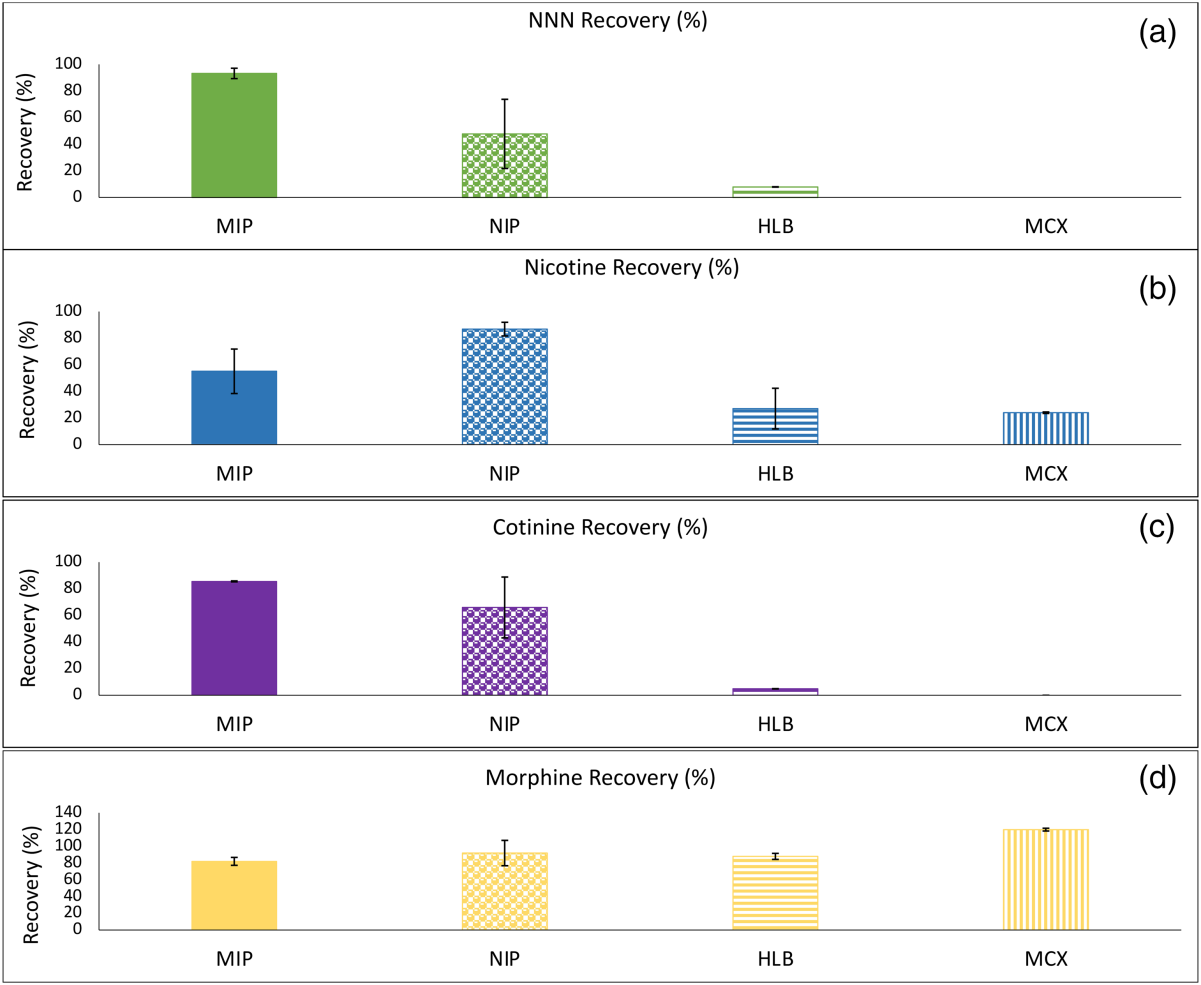
Average recoveries (mean ± SD, N = 3) of (a) *N*‐nitrosonornicotine (NNN), (b) nicotine, (c) cotinine and (d) morphine with the following solid‐phase extraction (SPE) cartridges: tobacco‐specific nitrosamine (TSNA) molecularly imprinted polymer (MIP) cartridges, nonimprinted polymer (NIP) cartridges, Waters Oasis hydrophilic–lipophilic balance (HLB) cartridges and Waters Oasis mixed‐mode cation exchange (MCX) cartridges. Analytes loaded onto the MIP and NIP were adjusted to a pH of 5.5 (NNN and cotinine) or 9.2 (nicotine and morphine) to reflect an ionization stated of 15%. For the HLB cartridges, samples were loaded under acidic conditions (pH 2.79 for NNN and cotinine and pH 5.5 for nicotine and morphine). For the MCX cartridges, samples were loaded under basic conditions (pH 10 for all analytes). For the MCX cartridges, NNN and cotinine eluted prematurely during the methanolic washing step and a portion of nicotine was also eluted during the washing step

**FIGURE 2:**
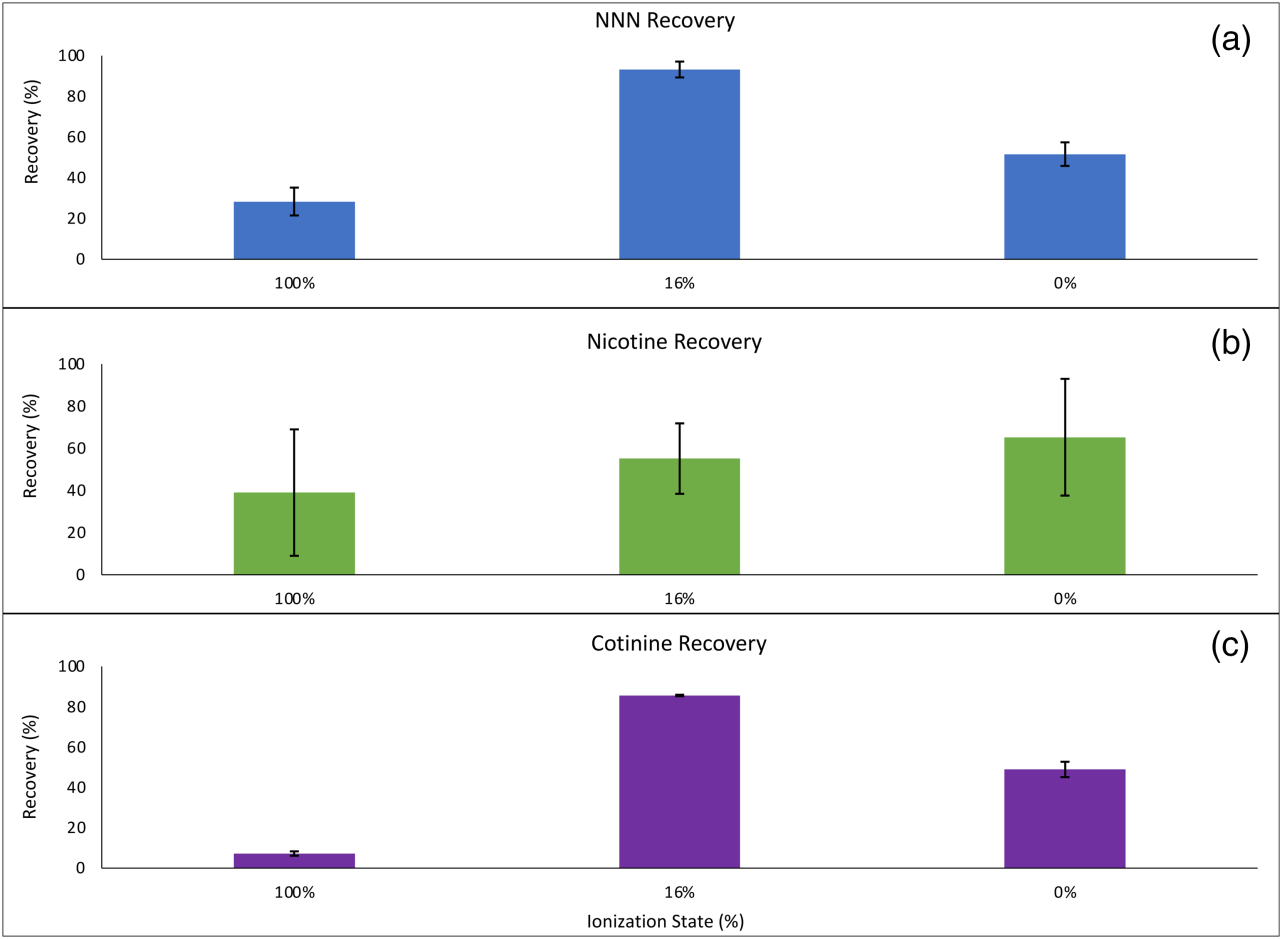
Effect on average recovery (mean ± SD, *N* = 3) of (a) NNN, (b) nicotine and (c) cotinine when the pH of the loading solvent was adjusted. NNN and cotinine were 100% ionized at a pH of 2.79, 16% ionized at a pH of 5.5 and 0% ionized at a pH of 10. Nicotine was 100% ionized at a pH of 5.5, 16% ionized at a pH of 9.2 and 100% ionized at a pH of 10

The SupelMIP™ TSNA cartridge recommends that samples are loaded at a pH of 5.5, when NNN is 16% ionized. At a pH of 5.5, the recovery of NNN with the MIP cartridge was 93.21 ± 3.90% (Figure 1). When adjusting the pH to be either 100% ionized (pH 2.79) or 0% ionized (pH 10), a drop in recovery was observed with NNN recoveries as low as 28.36 ± 7.23 and 51.64 ± 3.82%, respectively (Figure 2). With the NIP SPE cartridge, the recovery of NNN at a pH of 5.5 was 47.74 ± 26.00%. There was a statistical difference (*p* = 0.0092) in the average recoveries of NNN at pH 5.5 with the MIP and NIP cartridges. Compared with the traditional SPE methods, the average recovery of NNN with the hydrophilic–lipophilic based (HLB) SPE cartridge was 7.89 ± 0.16% (Figure 1). With the mixed‐mode cation exchange cartridge (MCX), NNN was prematurely eluted in the washing stage with average recovery 26.83 ± 6.08%, and was not present in the elution fraction. Recovery of NNN with the MIP cartridge compared with the traditional SPE cartridges was significantly different (*p* < 0.05).

### 3.2 Nicotine

At a pH of 9.2, when 16% of nicotine was ionized, the average recovery with the MIP cartridge was 55.13 ± 16.72% (Figure 1). At a pH where nicotine was 100% ionized (pH 5.5), recovery was 39.00 ± 30.00%, and when 0% ionized (pH 10), recovery was 65.28 ± 27.70% (Figure 2). The cross‐reactivity of the imprinted polymer for nicotine was calculated using the Equation 1,

(1) $$\frac{\text{Recovery}\;\left(\%\right)\;\text{of analyte}}{\text{Recovery}\;\left(\%\right)\;\text{of}\;\mathrm{NNN}}\times 100$$

where the recovery (%) of the analyte is the non‐TSNA tobacco alkaloid extracted with the MIP cartridge and the recovery (%) of NNN extracted with the MIP cartridge, which the imprinted polymer is designed for. Nicotine had a cross‐reactivity of 59.15% at a pH of 5.5.

Average recovery with the nonimprinted polymer was 86.84 ± 5.13%. There was a statistical difference (*p* = 0.0448) in the average recoveries of nicotine with the MIP cartridge and the NIP cartridge. With the traditional SPE cartridges, nicotine's average recovery was 27.10 ± 15.28% for the HLB cartridge and 24.03 ± 0.56% for the MCX cartridge (Figure 1). A portion of nicotine was prematurely eluted during the washing step of the MCX cartridge.

The variability of nicotine recovery with the MIP cartridge was improved when the elution solvent was adjusted to a more lipophilic solvent, consisting of 9:1 MtBE:THF. Recovery of nicotine at pH 5.5 under these conditions was 42.60 ± 1.73%.

### 3.3 Cotinine

Similar to NNN, cotinine was 16% ionized at a pH of 5.5, and had an average recovery of 85.60 ± 0.36% with the MIP cartridge (Figure 1). When cotinine was 100% ionized (pH 2.79) and 0% ionized (pH 10), its recoveries were 7.23 ± 1.09 and 48.91 ± 3.82%, respectively (Figure 2). Using Equation 1, cotinine had a cross‐reactivity with the MIP cartridge of 91.83% at a pH of 5.5. For the NIP SPE cartridge, cotinine had an average recovery of 65.99 ± 22.86%, and there was no statistical difference in the average recoveries of the MIP and NIP cartridges (*p* = 0.2316). With the traditional SPE cartridges, cotinine's average recovery with the HLB SPE cartridge was 4.87 ± 0.09% (Figure 1). Similar to NNN, cotinine was prematurely eluted in the washing step of the MCX cartridge, with average recovery being 32.82 ± 14.07%. There was a statistical difference (*p* < 0.05) between the recoveries of cotinine with the MIP cartridge and the traditional SPE cartridges.

Absolute recovery and matrix effects of cotinine in urine were calculated. Absolute recovery was determined by the measured response of the cotinine extract to an unextracted cotinine solution of the same concentration. Matrix effects were calculated by the measured response of a blank extract that was infused with cotinine prior to injection to a neat, unextracted cotinine standard of the same concentration. Absolute recovery of cotinine in urine was >85% for all three concentrations. Matrix effect measured at the middle concentration, 100 ng/ml, was −7.84% (Table 2).

**TABLE 2:** Recovery of cotinine at 10, 100, and 1,000 ng/ml in urine with matrix effects for middle concentration (*N* = 3)

Concentration (ng/ml)	Recovery (%) ± standard deviation	Matrix effects (%) ± standard deviation
10	97.05 ± 2.54	
100	107.84 ± 2.13	−7.84 ± 2.13
1,000	89.3 ± 8.49	

### 3.4 Morphine

At a pH of 9.2, morphine was 16% ionized and had an average recovery of 82.00 ± 4.73% (Figure 1) and a cross‐reactivity of 87.98% with the MIP cartridge. Average recovery with the NIP cartridge at the same pH was 91.93%. There was no statistical difference between the morphine recoveries with the MIP and NIP cartridges (*p* = 0.6216). With the traditional SPE cartridges, morphine had an average recovery of 87.97 ± 3.60% with the HLB cartridge and 119.88 ± 1.66% with the MCX cartridge. Only the MIP cartridge and the MCX cartridge had a statistically different recovery (*p* = 0.0380).

## 4 DISCUSSION

The theory behind molecularly imprinted polymers is that the selective interaction is governed by the size, shape and functional group chemistry of the target analyte with the imprinted cavity and functional monomer (Turiel & Esteban, 2020). Following this theory, analytes with similar structures and chemistries to the target analyte should be able to interact with an imprinted cavity under similar conditions. The imprinted polymer used in this study was designed for the extraction of four TSNAs in urine. Owing to the nature of the imprinted polymer being class selective for four analytes and having shown cross‐selectivity for urinary metabolite NNAL, (Boyd et al., 2007; Xia et al., 2014), it was hypothesized that other tobacco alkaloids and their metabolites could also be selectively extracted with the imprinted polymer.

### 4.1 Analyte recovery with the imprinted polymer

NNN was used as a positive control in this study. The recovery of NNN was compared against the recovery of the other three analytes with the MIP cartridge. Then, the recovery of NNN with the MIP cartridge was compared with a nonimprinted polymer and two traditional SPE techniques for NNN. It was therefore unsurprising that NNN had the highest recoveries with the MIP cartridge. Nicotine, which is a precursor to the TSNA NNK and to a lesser extent, NNN (Xia et al., 2014), had lower recoveries compared with NNN and residual standard deviation (RSD) values >15%, indicating high variability with the extraction. Nicotine also had the lowest cross‐reactivity value of the analytes used to characterize the polymer in this study. While NNN is partially derived from nicotine and both contain pyridine and pyrrole functional groups, nicotine lacks the nitrosamine group present on all TSNAs, which may be a site of interaction between the analyte and the functional monomer. The differing chemical properties between nicotine and NNN may further disrupt nicotine's interactions with the TSNA MIP cartridge. Nicotine has a higher p*K*_a_ of 8.58, compared with NNN's p*K*_a_ of 4.79 (Table 3). The manufacturer recommends that TSNAs be pre‐treated to a pH of 5.5, where about 16% of the TSNA is in its ionized state. When adjusting the pH of nicotine to meet those same conditions (pH 9.2), there was no improvement in the recovery of nicotine. Overall, the pH/ionization state of nicotine was not a driving force in its recovery, as seen with the amphetamine‐MIP in Murakami et al. (2018) (Figure 2). Second, the partition coefficient (log*P*) of NNN is −0.08, making it hydrophilic in nature whereas nicotine's log*P* is 0.72, and is more lipophilic. When the elution solvent was changed to more lipophilic solvents, such as the 9:1 MTBE:THF, the variability of nicotine's recovery decreased with RSD values <5%. Nicotine's average recovery, however, was still lower than that of NNNs, with recoveries <50% with the TSNA MIP cartridge. Other extraction techniques for nicotine in biological matrices such as SPE and micro‐solid phase extraction report >80% recoveries (Kataoka et al., 2009; Miller et al., 2010). The low recoveries of nicotine in this study did not warrant further attempts to utilize the selectivity of the TSNA MIP cartridge for nicotine in biological matrices.

**TABLE 3:** Chemical information for *N*‐nitrosonornicotine (NNN), nicotine, cotinine and morphine

Analyte	Structure	Molecular weight (g/Mol)	p*K*_a_	Log*P*
NNN	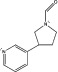	177.207	4.79	−0.08
Nicotine	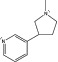	163.263	8.58	0.72
Cotinine	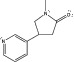	176.219	4.79	−0.30
Morphine	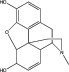	285.338	8.21	0.80

Cotinine, like NNN, also had recoveries >80%. Cotinine also had the highest cross‐reactivity value with the TSNA MIP cartridge (Figure 1). While cotinine lacks a nitrosamine group, it does have a carbonyl group present on the pyrrole that could potentially interact with the functional monomer. Considering that cotinine has similar chemical properties to NNN (the same p*K*_a_ and site of ionization), it follows that cotinine had similar, high recoveries with the TSNA MIP. Cotinine also followed the same recovery trend that NNN displayed when the pH of the sample was adjusted (Figure 2). The leading difference between cotinine and NNN is that, like nicotine, cotinine and NNN have differing log*P* values. Cotinine, as a urinary metabolite of nicotine, has a log*P* of −0.32 and is more hydrophilic than NNN. While not investigated in this study, it is possible that adjusting the elution solvent for cotinine could lead to greater recoveries. Cotinine's successful recovery in urine (>85%) for all three concentrations indicates that it can be reliably extracted from biological samples with the TSNA MIP. Coupled with the previously reported successful co‐extraction of NNAL with the TSNA MIP, the simultaneous extraction of cotinine with the TSNAs and their metabolites warrants further exploration. Urinary cotinine levels are often correlated with NNN and the urinary metabolite of NNK, NNAL, for total nicotine equivalence (Marques et al., 2021; Xia et al., 2005). The TSNA MIP cartridges could potentially simultaneously extract cotinine and TSNAs while avoiding the matrix challenges that have been encountered in other simultaneous extractions (Kotandeniya et al., 2015; Luo et al., 2021).

The extraction of morphine with the TSNA MIP cartridge was used to assess the recovery of an analyte that was not a tobacco alkaloid and was structurally different. Morphine was selected because of its similar p*K*_a_ and log*P* values to nicotine (Table 3), and it was initially theorized that morphine would exhibit the same variability observed with nicotine. However, morphine had >80% recovery with the TSNA MIP and low variability (RSD < 15%). While morphine most likely does not fit into the imprinted cavities, it could interact with the MIP cartridge through surface, nonspecific interactions. Nonspecific interactions are formed during the polymerization of the imprinted polymer, owing to the presence of excess functional monomers that do not form bonds with the template (Vasapollo et al., 2011). Morphine's interactions with the excess functional would lead to the high recoveries. In future studies, it would be beneficial to determine if morphine's presence would affect the extraction recovery of the TSNAs.

### 4.2 Analyte recovery with the nonimprinted polymer

The NIP serves as a control for the MIP. The NIP is created in the absence of a template, preventing the formation of imprinted cavities (Pichon et al., 2019). Therefore, all interactions with the NIP are considered nonspecific. The recovery of NNN with the NIP cartridges resulted in <50% recovery and high variability (RSD > 15%). The low recovery and high variability with the NIP in comparison with the TSNA MIP cartridge suggest that specific interactions with the imprinted region of the MIP aid in the retention and recovery of the TSNAs. Cotinine followed a similar trend with the NIP cartridge (Figure 1). Nicotine, however, had >80% recovery with low variability (RSD < 10%) with the NIP cartridge, without having to make changes to the elution solvent. This suggests that the imprinted cavity has an effect on the the retention and recovery of nicotine with the TSNA MIP cartridge. Morphine's similar recovery with the TSNA MIP and NIP cartridges further suggests that its recoveries are due to nonspecific interactions with the excess functional monomer. It should also be noted that all experiments in this study were completed in buffered water in the absence of matrix. Recovery of the analytes in the presence of matrix effects should be studied further.

### 4.3 Analyte recovery with traditional SPE cartridges

Extraction of the analytes using traditional SPE cartridge methods designed for TSNAs were compared with the recovery of the analytes with the TSNA MIP cartridges. The HLB cartridges are designed with hydrophilic and lipophilic copolymers, acting as a reverse‐phase SPE cartridge and allowing for a wide range of analytes to be extracted with the cartridge (Oasis Sample Extraction Product Brochure, 2017). The MCX cartridge is a reverse‐phase sorbent derived from the HLB copolymers and is designed for base analytes. The extraction protocols used in this study were optimized for the extraction of TSNAs (Kavvadias et al., 2009; Wu et al., 2003).

Previous reports stated that TSNAs have low recoveries with traditional SPE cartridges and require multiple extractions to efficiently remove matrix interferents. (Boyd et al., 2007; Wu et al., 2003). Using previously published methods for the extraction of NNN, the HLB SPE recoveries for both NNN and cotinine were <10%. Nicotine had recoveries <30% with the same method while morphine had recoveries similar to those with the TSNA MIP and NIP cartridges. With the MCX cartridges, NNN and cotinine eluted during the methanolic washing step. The early elution with the MCX cartridge could be due to the fact that the analytes were loaded in an unionized state. Nicotine had recoveries <30% with the MCX cartridges, and a fraction of nicotine also eluted during the washing step. The low recoveries of nicotine with the traditional SPE cartridges are probably due to the fact that the extraction methods were designed for the extraction and recovery of TSNAs. Previous literature using SPE and micro‐solid phase extraction techniques for the recovery of nicotine in urine yielded >80% recoveries (Kataoka et al., 2009; Miller et al., 2010). Morphine had a >100% recovery with the MCX cartridge, possibly caused by an analyte co‐eluting at the same wavelength on the instrument. The >80% recoveries with morphine for all four cartridges further suggest that morphine's high recovery with the MIP TSNA cartridge was caused by nonspecific interactions.

## 5 CONCLUSIONS

An extensive characterization of the cross‐selectivity of the TSNA MIP cartridges was performed in this study. NNN, which the TSNA MIP was designed for, had the highest recoveries of all the analytes extracted. The TSNA MIP's superior performance for the extraction of NNN compared with traditional SPE cartridges suggests that MIPs can improve the extraction recovery. Nicotine, despite being a precursor to TSNAs, had highly variable recoveries, possibly owing to the differing structural and chemical properties between the precursor and the TSNAs. Cotinine had similar recoveries with NNN, most likely owing to its similar chemical properties to the TSNAs. This study showed that the MIP's cross‐selectivity can be utilized for the selective extraction of cotinine in urine. Despite morphine's high (>80%) recovery with the TSNA MIP, the similar recoveries with the NIP and traditional SPE cartridges suggests that morphine's affinity for the polymer was due to nonspecific interactions. The utilization of the TSNA's cross‐selectivity with other tobacco biomarkers can allow for the simultaneous extraction and identification of multiple biomarkers with one, simple sample preparation technique.
